# Citywide preparedness for a pandemic: A cross-sectional survey of knowledge, attitudes, and practices about respiratory infection prevention in Bogotá, Colombia

**DOI:** 10.7705/biomedica.5526

**Published:** 2020-11-15

**Authors:** Jorge Alberto Cortés, Pilar Espitia, Yuliet Liliana Rosero-Lasso

**Affiliations:** 1 Departamento de Medicina Interna, Universidad Nacional de Colombia, Bogotá, D.C., Colombia Universidad Nacional de Colombia Departamento de Medicina Interna Universidad Nacional de Colombia BogotáD.C Colombia; 2 Hospital Universitario Nacional de Colombia, Universidad Nacional de Colombia, Bogotá, D.C., Colombia Universidad Nacional de Colombia Universidad Nacional de Colombia BogotáD.C Colombia; 3 División de Salud Pública, Secretaría Distrital de Salud, Bogotá, D.C., Colombia Secretaría Distrital de Salud BogotáD.C Colombia

**Keywords:** Coronavirus infections, health knowledge, attitudes, practice, Colombia, respiratory tract infections/prevention and control, infection control practitioners, health personnel, infecciones por coronavirus, conocimientos, actitudes y práctica en salud, infecciones del sistema respiratorio/prevención y control, profesionales para control de infecciones, personal de salud

## Abstract

**Introduction:**

Healthcare personnel plays an important role in the prevention of acute respiratory infections in hospital settings.

**Objective:**

Our aim was to establish the level of knowledge about respiratory virus infections and the attitudes and practices among healthcare workers, leaders of infection control committees in hospitals of Bogotá, Colombia.

**Materials and methods:**

We used a self-administered questionnaire of 28 items during the monthly meeting sponsored by the local health authority. "Yes or no" and "true or false" questions were applied to measure knowledge. Attitudes and practices were measured with a Likert-type scale according to the agreement degree.

**Results:**

We surveyed 70 healthcare workers. Respondents demonstrated a good level of knowledge as 80% of them answered correctly more than five questions. A total of 54.4% showed a low degree of agreement when asked if their institutions have the policy to stay home when they are sick with respiratory symptoms and 67.1% never or rarely remain at home under such conditions.

**Conclusion:**

Healthcare worker leaders of infection control committees in Bogotá's hospitals have adequate knowledge about the prevention of seasonal respiratory viruses. There is a need for implementing urgent sick leave policies as a measure to prevent the spread of potential coronavirus infections in hospitals.

## Resumen

**Introducción.:**

El personal de salud juega un papel importante en la prevención de la diseminación de los virus respiratorios en los hospitales.

**Objetivo.:**

Establecer el nivel de conocimiento y determinar las actitudes y prácticas en relación con los virus respiratorios entre los encargados de los comités de infecciones de los hospitales de Bogotá.

**Materiales y métodos.:**

Los participantes respondieron una encuesta de 28 ítems durante una de las sesiones mensuales del comité de infecciones de la ciudad. Se midió el conocimiento y se formularon preguntas sobre las actitudes y las prácticas utilizando una escala de tipo Likert para evaluar la conformidad.

**Resultados.:**

Se encuestaron 70 trabajadores de salud. Los participantes tenían un buen nivel de conocimiento, ya que el 80 % de los respondientes tuvieron cinco o más respuestas correctas. El 54,4 % mostró un bajo nivel de conformidad en cuanto a si sus instituciones tenían una política de quedarse en casa en caso de síntomas respiratorios y 64,1 % nunca o casi nunca se queda en casa cuando presenta dichos síntomas.

**Conclusión.:**

Los trabajadores de la salud que encabezan los comités de infecciones de los hospitales de Bogotá tienen un adecuado conocimiento de la prevención de los virus respiratorios. Deben implementarse políticas de quedarse en casa para el personal con síntomas gripales, con el fin de prevenir la potencial diseminación de virus en los hospitales.

Hospital-acquired respiratory viral infections can result in morbidity and mortality of hospitalized patients ^1^. In Colombia, viral circulation has the typical pattern of tropical countries as it occurs throughout the year but increases in the rainy seasons (April-June and September-December) demanding preparedness for a "viral season" at least twice a year. Influenza is the most commonly identified virus ^2^ but others also circulate regularly.

In December, 2019, an outbreak of severe acute respiratory syndrome coronavirus 2 (SARS-CoV-2) infection was reported in Wuhan, China, which rapidly spread across this and other countries ^3^^-^^5^ including American countries. SARS-CoV-2 can be transmitted effectively among humans and exhibits high potential for a pandemic ^6^. At the beginning of April, 2020, the World Health Organization (WHO) reported confirmed cases in the majority of American countries: the United States, Canada, Brazil, México, and Ecuador ^7^ suggesting ongoing circulation in the region. Colombia has been no exception and the pandemic peak will coincide with that of respiratory virus infections during the first rainy season of the year.

Bogotá, the largest city in the country, with over 7.4 million inhabitants, has the largest healthcare infrastructure. Healthcare personnel plays an important role in the prevention of acute respiratory infections in hospital settings ^8^, so it is essential that they have adequate knowledge about these infections and they should exert leadership in the strategies aimed at reducing the risk of hospitalized patients acquiring respiratory infections.

The present study aimed to determine the level of knowledge about respiratory virus infections and the attitudes and perceptions of healthcare worker leaders in infections control committees of hospitals in Bogotá and to establish a baseline to define educational priorities for the upcoming SARS-CoV-2 pandemic.

## Materials and methods

### Population

We conducted a cross-sectional study among leaders of infection control committees of hospitals in Bogotá, Colombia. Bogotá is the largest city in the country, with over 7.4 million inhabitants (without the surrounding small towns), and accounts for the largest number of healthcare institutions (69 second or third level hospitals).

We administered a questionnaire on February 14^th^, 2020, during the monthly meeting sponsored by the city's local health authority among those in charge of the infection control committees. No sample was taken from the assistants and the questionnaire was offered to all the participants. The respondents are responsible for guiding the decisions of the infection control committees in each second or third level hospital, establishing policies and practices related to infection control and aiding, together with public health authorities, in the implementation of surveillance, prevention, and control strategies of respiratory tract infections in the institutions during the "rainy season" in the city.

### Survey instrument

A self-administered survey with 28 questions in Spanish was applied. The questionnaire comprised four main sections on: (A) demographic characteristics, (B) knowledge, (C) attitudes, and (D) practices of healthcare worker leaders of infection control committees regarding prevention of respiratory virus infections.

To measure knowledge, we asked "yes or no" and "true or false" questions. For questions on attitudes and practices, participants' agreement with a given statement was measured on a 5-point Likert scale ("strongly agree" "agree" "uncertain", "disagree", and "strongly disagree" or "never", "sometimes", "often", "most of the time"' and "always").

The knowledge section included questions about viral transmission, prevention of viral infection through hand hygiene, gloves, patient identification, use of masks, and seasonal circulation of viruses in the country.

The attitudes section included the point of view of the respondent about sick leave policies, respiratory patient identification and education, and isolation practices.

The practices section included habits or uses of isolation and protection, hand hygiene, and patient education.

### Data analysis

The data obtained were stored in a database using Microsoft Office Excel, version 2019, and analyzed with Stata™, version 16. The results were reported in absolute values, proportions, and measures of central tendency (medians and interquartile ranges). Comparisons were done with the Wilcoxon test between continuous variables and with the chi-square test for proportions.

### Ethical aspects

The questionnaire was designed to be individual and anonymous and participation was voluntary so informed consent was not specifically obtained. The data were kept confidential and the results did not individually identify the respondents.

## Results

We surveyed 70 healthcare worker leaders from infection control committees in city hospitals. The majority (84.3%) of the respondents were women. Occupations included nursing assistant, nurse, doctor, and others. A total of 81.4% of the respondents worked directly with the infection committee of their institution. Table 1 shows their demographic data.

**Table 1:** Demographic characteristics of survey respondents (N=70)

Characteristic	n (%)
Sex	
Female	59 (84.29)
Age (years)	
Median	37
Less than 30	17 (27.4)
31-46	33 (53.2)
More than 46	12 (17.2)
Education level	
Postgraduate	52 (74.3)
University	11 (15.7)
Technical	7 (10.0)
Occupation	
Nursing assistant	6 (8.57)
Nurse	47 (67.14)
Physician	6 (8.57)
Pharmaceutical chemist	1 (1.43)
Other	10 (14.29)
Infection committee (directly)	
Yes	57 (81.43)
Type of institution	
Private	57 (81.43)
Public	13 (18.57)
Institution level	
Level I	5 (7.14)
Level II	14 (20.0)
Level III	19 (27.14)
No information	32 (45.71)
Time working in healthcare area (years)	
Median	10
1-7	29 (42.7)
8-14	15 (22.1)
15-21	12 (17.7)
More than 21	12 (17.7)
Time working in the institution (years)	
Median	3
0-5	45 (70.3)
6-10	6 (9.4)
More than 10	13 (21.3)

### Knowledge

**Figure 1:**
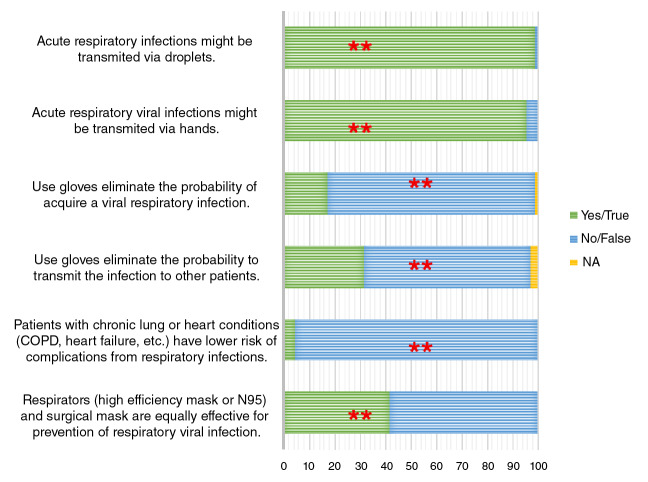
Knowledge regarding respiratory viral infections (N=70). The figure shows the correct answer.

Figure 1 shows the results obtained for the knowledge questions. Interestingly, we found that 58.0% (95% CI: 45.9-65.2) of the respondents considered that high-efficiency respirators and surgical masks are not equally effective for respiratory viral infection prevention.

Regarding the question about the season of respiratory virus circulation in Bogotá, most respondents (64.1%, 95% CI: 51.4-71.0) knew that the circulation of these viruses occurs throughout the year; however, there is an important percentage that had incorrect knowledge about this issue.

Eighty percent of respondents answered more than five questions correctly. The median number of correct answers was 6 for nurses, 5.5 for physicians, and 4.5 for nurse assistants (p=0.016 for the comparison between nurses and the assistants). Health care workers in III level hospitals had higher scores of correct answers (6 for the third level vs 5 for the second level hospitals, p=0.036). No effect on the scores was seen regarding the number of years of work.

### Attitudes

**Figure 2:**
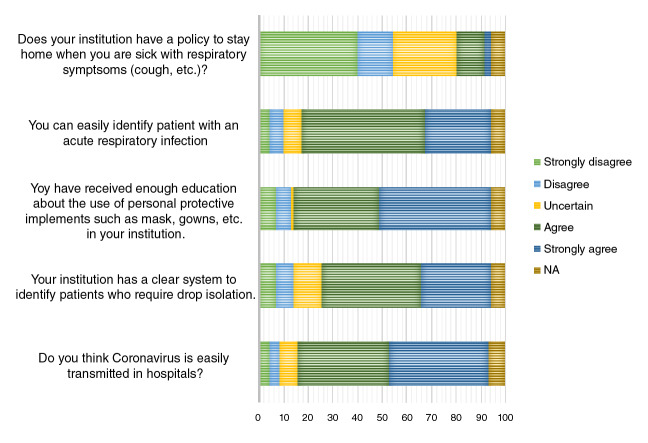
Attitudes regarding transmitted in hospitals?

Figure 2 shows the results regarding the attitudes of the respondents. Most of the questions on attitudes were answered with a high degree of agreement. It is interesting to note that 57.5% of the respondents (95% CI: 39.2 - 81.0) had a low degree of agreement about the leave of absence policy in institutions when they have respiratory symptoms.

Most respondents reported that most of the time or always they perform hand hygiene before entering or exiting a patient's room with 86.4% (95% CI: 75.6-92.8) agreement in both cases. There were no differences in the degree of agreement between different occupations or complexity levels in the hospitals.

### Practices

**Figure 3:**
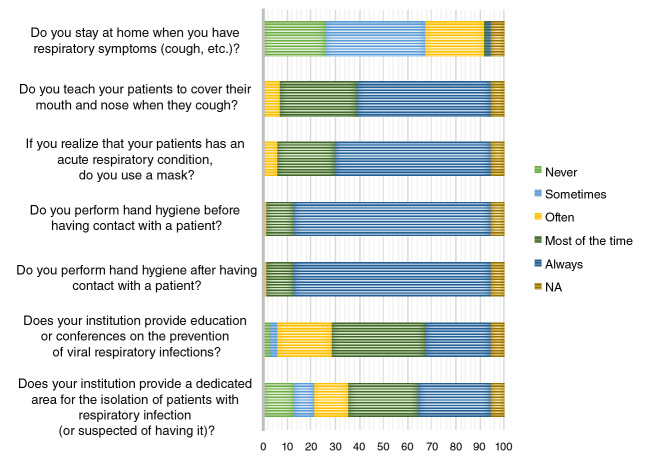
Practices regarding respiratory viral infections (N=70) NA: Question not answered

Figure 3 shows the results regarding the practices of the respondents. Most of them reported that they sometimes perform the practices shown in figure 3. However, most respondents (71.2%, 95% CI: 50.1-95.7) stated that they never or only sometimes remain at home when they have respiratory symptoms. There were no differences in the practices between occupations or levels of complexity.

## Discussion

Our results show that the leaders of infection control committees in Bogotá's hospitals have adequate knowledge about the prevention of seasonal respiratory viruses which evidences that they may be adequately prepared to face the dissemination of these viruses during the rainy season and that there is a baseline preparation for the SARS-COV2 pandemic. This fact is important since leaders are required to have an important level of knowledge and to be capable of systematically transmitting that knowledge to their coworkers and peers to ensure they have adequate knowledge and skills to prevent viral respiratory infection at hospitals ^9^.

Despite finding an adequate level of knowledge and skills, there are also some deficiencies in terms of basic prevention measures, such as hand hygiene, which is widely known as a very effective prevention measure for respiratory infections ^10^^-^^12^. It seems to be an important but endless task in infection control and prevention. Other misconceptions, such as the efficacy of respirators (N95) versus the traditional mask for respiratory infection prevention should be clarified among the personnel surveyed since it is known that there is no significant difference between the use of one or the other, even in the prevention of the influenza virus ^13^. Current CDC guidelines recommend the use of medical respirators only for healthcare personnel that needs protection from both airborne and fluid hazards ^14^. In a pandemic, the risk of a capacity crisis might put in danger health care workers due to a shortage of key personal protection elements, so the correct use of some of them is fundamental to prevent such shortage.

Responses about sick leave for healthcare workers show that there is a lack of regulation regarding policies to stay at home when they have respiratory symptoms. Although it has been shown that sick leave policies among healthcare workers are important in reducing the transmission of infections in the hospital, many hospitals in the United States do not have such policies to ensure restriction on direct patient care by sick hospital staff ^15^.

In the case of Colombia, the lack of these policies respond to several situations: no clear guidelines on the matter by the country's official entities, as well as economic reasons among the healthcare personnel since payment might be stopped or diminished because of local social security regulations ^16^. This sick leave policy and a clear understanding of the role of healthcare workers in virus transmission are important to limit the number of infections in the hospitals both in healthcare workers and in patients.

Our study has limitations. First, the survey was not validated before its application. Second, it was applied only to infection committee leaders as an indirect measure of the entire population of healthcare personnel in the hospitals of Bogotá to evaluate whether the city was prepared for an eventual SARS CoV-2 pandemic.

Despite these limitations, we consider that our findings can stimulate the design of interventions to strengthen knowledge, modify attitudes, and optimize practices regarding the prevention of acute respiratory infections and to promote the implementation of policies such as sick leave as a potential intervention to prevent the spread of acute respiratory infections in hospitals.
